# The Fission Yeast XMAP215 Homolog Dis1p Is Involved in Microtubule Bundle Organization

**DOI:** 10.1371/journal.pone.0014201

**Published:** 2010-12-02

**Authors:** Hélio Roque, Jonathan J. Ward, Lindsay Murrells, Damian Brunner, Claude Antony

**Affiliations:** European Molecular Biology Laboratory, Cell Biology and Biophysics Program, Heidelberg, Germany; Texas A&M University, United States of America

## Abstract

Microtubules are essential for a variety of fundamental cellular processes such as organelle positioning and control of cell shape. *Schizosaccharomyces pombe* is an ideal organism for studying the function and organization of microtubules into bundles in interphase cells. Using light microscopy and electron tomography we analyzed the bundle organization of interphase microtubules in *S. pombe*. We show that cells lacking ase1p and klp2p still contain microtubule bundles. In addition, we show that ase1p is the major determinant of inter-microtubule spacing in interphase bundles since *ase1* deleted cells have an inter-microtubule spacing that differs from that observed in wild-type cells. We then identified dis1p, a XMAP215 homologue, as factor that promotes the stabilization of microtubule bundles. In wild-type cells dis1p partially co-localized with ase1p at regions of microtubule overlap. In cells deleted for *ase1* and *klp2*, dis1p accumulated at the overlap regions of interphase microtubule bundles. In cells lacking all three proteins, both microtubule bundling and inter-microtubule spacing were further reduced, suggesting that Dis1p contributes to interphase microtubule bundling.

## Introduction

The formation of microtubule (MT) arrays and the organization of individual MTs is essential for diverse cellular functions such as spindle biogenesis [1], regulation of neuronal growth [2] and organelle positioning [3], [4]. Detailed knowledge of the spatial distribution of microtubules is also necessary for modeling of MT assemblies [5].

The fission yeast *Schizosaccharomyces pombe* is a well-established model organism for studying the control of cell polarity, MT dynamics and spindle assembly [6], [7], [8]. During interphase, most MTs are associated with each other in bundles, although a few individual MTs are also present [9]. Since it is difficult to unambiguously distinguish between single MTs and bundled MTs using fluorescence microscopy, both arrangements are generally termed “interphase microtubule assemblies” (IMAs) [6], [10]. IMAs are essential for determining the sites of growth in *S. pombe* [7] and defects in their organization lead to cells with abnormal morphologies [8], [11], [12], [13].

A typical interphase cell contains between 3 and 6 dynamic IMAs [10]. Each IMA is composed of between 2 and 9 MTs [9]. A typical IMA possesses a relatively stable, centrally located medial region, with anti-parallel MTs overlapping at their minus ends [9] with the distal plus ends undergoing repeated cycles of MT growth towards and shrinkage away from the cell poles [10], [11], [14]. Such organization is important to ensure that MT plus tips are orientated towards both cell poles where they mediate the deposition of polarity markers [7].

IMA formation, organization and maintenance is achieved by four main processes: the targeting of MT nucleation to preexisting MTs; the bundling of MTs; the confinement of MT overlaps to MT minus end regions; and the localized regulation of MT plus end dynamics.

Targeting of interphase MT nucleation is mediated by the microtubule-associated proteins (MAPs) mto1p (also known as mbo1 or mod20) and mto2p. These proteins recruit the gamma tubulin ring complex (γTuRC) to several sites in the cell including the lattice of pre-existing MTs [15], [16], [17], [18], [19], [20]. In addition, mto1p recruits the transforming acidic coil-coiled (TACC) protein Mia1p/Alp7p to the overlap regions where it was proposed to contribute to MT cross-linking and the stabilization of newly-formed MT overlaps [21]. When a new microtubule is nucleated dendritically along the lattice of an existing MT, ase1p, a member of the MAP65/PRC1/ASE1 protein family bundles the MTs in an anti-parallel orientation [22], [23]. Klp2p, a member of the minus end-directed kinesin–14 protein family [24] then slides the “daughter” MT towards the minus end of the pre-existing one [23] in this way confining the overlap regions to the minus-ends of the MTs. Klp2p has also been shown to bundle parallel MTs in a minimal *in vitro* system [25].

Finally, the global positioning of MT overlaps is achieved by spatial regulation of MT plus end dynamics. Shrinking MT plus ends can be “rescued” when they reach the central overlap regions by peg1p/cls1p, a CLASP-type protein that binds to ase1p [26], [27]. Conversely, the growing MT plus ends switch to shrinkage, an event termed catastrophe, almost exclusively upon reaching the cell ends. This is thought to occur mainly due to compressive forces created by the growing MTs when pushing against the cell cortex. This effect is enhanced by the action of klp5p and klp6p. These two proteins are members of the kinesin-8 family that from a heterodimer that also exerts a MT length-dependent depolymerizing activity on interphase MTs [28], [29].

IMAs organization is also dependent on the regulation of MT dynamics by several protein families. One of the most intensely studied families includes the Dis1/XMAP215/chTOG proteins. These proteins contain a variable number of tumour-overexpressed gene (TOG) domains at their N-termini. Members of the Dis1/XMAP215/chTOG family are present in all known eukaryotic organisms and have been shown to act as processive microtubule polymerases *in vitro* [30], which leads to a variety of effects such as promotion of microtubule growth, shrinkage or stabilization in different *in vivo* contexts [31]. *S. pombe* is so far the only organism in which two members of the Dis1/XMAP215 have been found present: Alp14p and Dis1p [32], [33]. Dis1p has been shown to bind to MTs *in vitro* and *in vivo* and both proteins are necessary for proper chromosome segregation [33], [34], [35]. In addition dis1p is localized to kinetochores during prophase in an interaction that is dependent of phosphorylation by cdc2, while its localization to spindle MTs during anaphase is dependent on an unknown phosphatase [36]. The budding yeast dis1p homolog, Stu2 has recently been proposed to recruit free tubulin dimers to growing MT plus ends [37], [38].

Our knowledge of MT bundle formation and organization is based mainly on the analysis of fluorescent imaging of wild type and mutant cells and on modeling. However, fluorescence microscopy does not provide the necessary resolution to reveal the ultrastructural details of MT bundles, namely the number of MTs per bundle, MT orientation within the bundle, and the inter-MT spacing (the distance from MT wall to MT wall). These parameters are however essential for the proper description and interpretation of mutant phenotypes and for modeling.

Electron tomography (ET) is a powerful method for the three-dimensional analysis of cell architecture and in particular for studying cytoskeleton assemblies [39]. The method has previously been used to reconstruct the entire array of IMAs from a full cell volume in fission yeast [40]. In this study we used ET to analyze IMAs in cells containing deletions of the *ase1* and/or *klp2* genes. We show that cells lacking ase1p and klp2p still form IMAs with overlapping MTs suggesting that additional bundling proteins contribute to the formation of IMAs. Finally, we identify Dis1p, one of the two *S. pombe* XMAP215 homologues, as a novel MT organizing factor which complements ase1p MT bundling function in interphase. This suggests that dis1p could act as a bundler in the organization of interphase MT arrays.

## Results

### MTs bundle in the absence of ase1p and klp2p

**Figure 1:**
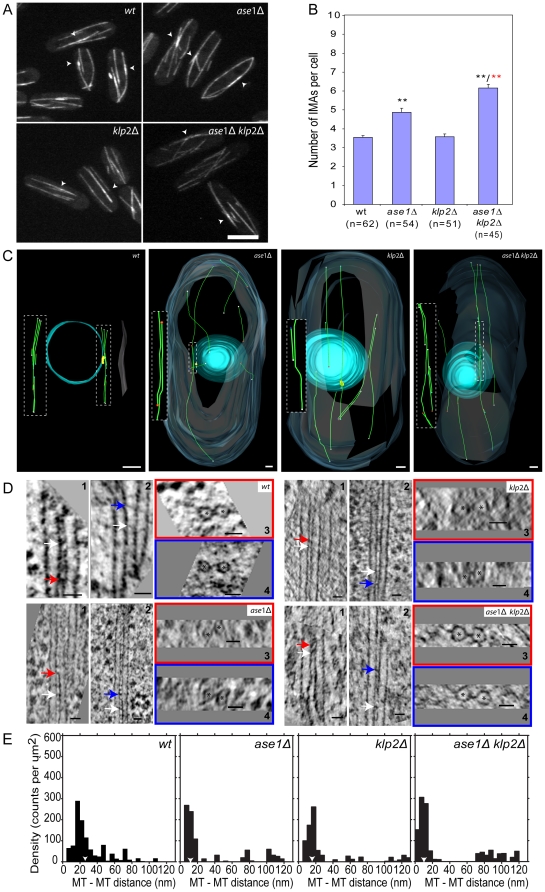
Bundled MTs are present in *ase1Δ klp2Δ* cells. A. Maximum projection of GFP-tubulin labeled cells where brighter fluorescence regions (arrowheads) are visible in wild-type and the different mutants. B. Number of IMAs per cell for wild-type and the different mutants. *p<0.05; **p<0.001 (mean ± SEM). Black asterisk are in relation to wild-type and red asterisks to *ase1Δ*. C. Models obtained from selected cells that are representative of wild-type and each mutant cytoskeleton organization. All the volumes show an inset of the dotted square where the MT bundle is isolated and rotated for better view. MT bundles are associated or non-associated with the SPB (yellow volume). MT ends structures are represented in colored spheres: red (capped structure), blue (blunt structure), cyan (open structures) and white (undetermined structure). Central volume represents the nucleus and surrounding volume represents the plasma membrane (equal pattern to all model images). See also Figure S1A and Movies S1–S3. D. Selected longitudinal tomographic sections of two adjacent MTs (asterisks) (1 and 2) showing electron-dense bridges (arrows). Cross-section of 1 and 2, respectively, in the plane of red and blue arrows. (E) Nda analysis shows that *ase1Δ* mutants have significantly smaller inter-MT distances than wild-type while *klp2Δ* cells only show a slight spacing reduction (arrowheads indicate the peak mean). Bars: 5 µm in A; 500 nm in C and 25 nm in D.

Previous studies on *ase1*-deleted (*ase1*Δ) cells suggested that MT overlap regions are essentially absent [22], [41]. We further analyzed IMAs in *ase1Δ* cells expressing GFP-tagged tubulin (GFP-tubulin) [42] and observed a 37% increase in the total number of IMAs (Figure 1A and B). We also observed a 30% decrease in the number of regions in IMAs containing fluorescence intensity twice or more that of single MTs (hereafter referred to as regions of increased fluorescence in order to distinguish these from the MT overlaps observed with electron tomography) (Table 1). The persistence of regions of increased fluorescence suggests that MT bundling is decreased but not abolished in *ase1*Δ cells [22], [41]. To confirm the existence of MT overlap regions we generated 3D models from tomograms of *ase1Δ* cells. In the reconstructed cell volumes we observed an increase in the number of single MTs compared to wild-type cells (Table 2). Despite this increase, we found regions of MT overlap in 8 out of 9 reconstructed cell volumes demonstrating that MT bundling occurs in the absence of ase1p (Figure 1C and Table 2).

**Table 1:** Number of regions of increased IMA fluorescence.

	*Regions of increased IMA fluorescence*	
	Average per cell ± SEM	n
*wild-type*	3.01±0.11	76
*ase1Δ*	2.1±0.12***	87
*klp2Δ*	2.48±0.11**	75
*ase1Δ klp2Δ*	1.37±0.12***	76
*dis1Δ*	2.16±0.09***	85
*ase1Δ dis1Δ*	1.21±0.12***	84
*ase1Δ dis1Δ klp2Δ*	1.72±0.13***	67
*81nmt1-dis1*	3.4±0.14*	70
*81nmt1-dis1 ase1Δ*	2.24±0.11***	68

*p<0.05;

**p<0.01;

***p<0.001 in relation to wild-type.

**Table 2:** Number of IMAs and MTs in the reconstructed EM volumes.

	*wild-type* *	*ase1Δ*	*klp2Δ*	*ase1Δ klp2Δ*	*dis1Δ*	*ase*1Δ *dis1*1Δ	*ase*1Δ *klp*2Δ *dis1*1Δ
N. of volumes	4	9	5	5	3	3	3
Total reconstructed volume (µm^3^)	ND	143.93	109.91	67.68	34.79	30.79	31.45
Single MTs per reconstructed volume	0.8±0.96n = 3	3.1±1.5n = 28	1.8±0.9n = 11	3.6±2.4n = 18	0	4.3±2.3n = 13	4.3±2.1n = 13
Bundles per volume	3±0.82n = 12	1.89±0.9n = 17	3.5±1.4n = 21	1.2±1.1n = 6	1.7±1.6n = 5	0.7±1.6n = 2	1.0±0.0n = 3
MTs per bundle	4.4±2.6	4±2	2.5±1.1	3.3±2.3	4±0	2±0	2.7±1.2

*Adapted from [9].

To test the possibility that the residual MT bundling observed in ase1p is mediated by klp2p [25], [43], [44], [45] we analyzed MTs in *klp2* deleted cells (*klp2Δ*) and cells deleted for both *ase1* and *klp2* (*ase1Δklp2Δ*). Fluorescence microscopy images of *klp2Δ* cells expressing GFP-tubulin showed that these cells contain a similar number of IMAs to wild-type cells (Figure 1A and B). The number of regions with increased fluorescence (Table 1) was decreased by 18% showing that MT bundling is affected by the lack of klp2p (Figure 1A and B). ET analysis confirmed that the number of MTs per bundle but not the number of bundles per reconstructed volume was altered in *klp2Δ* cells (Figure 1C and Table 2).

*Ase1Δklp2Δ* cells expressing GFP-tubulin showed a 73% increase in the number of IMAs indicating that bundling is strongly affected (Figure 1A and B). However, regions of increased fluorescence were present in the IMAs of these cells, again suggesting the presence of MT bundles (Table 1). We further analyzed these putative MT bundles by ET. In three out of five reconstructed *ase1Δklp2Δ* cell volumes we found bundles (Figure 1C and Table 2) showing that IMAs are maintained in the absence of ase1p and klp2p. Thus, an additional bundling activity exists independently of ase1p and klp2p.

The formation of electron-dense bridges that cross-link bundled MTs was documented for ase1p homologues [46], [47]. Similar electron-dense bridges were found in budding and fission yeast cells, but they have not yet been attributed to a specific protein [9], [48], [49], [50]. We generated dual axis tomograms to visualize electron-dense bridges in wild-type and mutant cells. In cells lacking ase1p and klp2p electron-dense bridges were observed between adjacent MTs (Figure 1D), which further supports the presence of an additional, specific MT bundling activity.

We further characterized the MT bundles with a “Neighbor Density Analysis” (Nda) to quantify the inter-MT spacing [48]. The reported values for wild-type cells are a preferred distance peak of 25 to 30 nm (mean at 26.2 nm; Figure 1E) [9]. *klp2Δ* cells showed a 21% reduction of the inter-MT spacing with a peak at 15–25 nm (mean at 20.8 nm) (Figure 1E). The major density peak for the global inter-MT distance in *ase1Δ* and *ase1Δklp2Δ* cells was even lower than in wild-type (∼55% less), peaking between 5–15 nm (mean at 11.8 nm) and 0–15 nm (mean at 12.6 nm), respectively (Figure 1E). Our data suggests that ase1p sets the higher spacing of bundled microtubules in wild-type cells.

### Dis1p is a putative MT bundling factor

**Figure 2:**
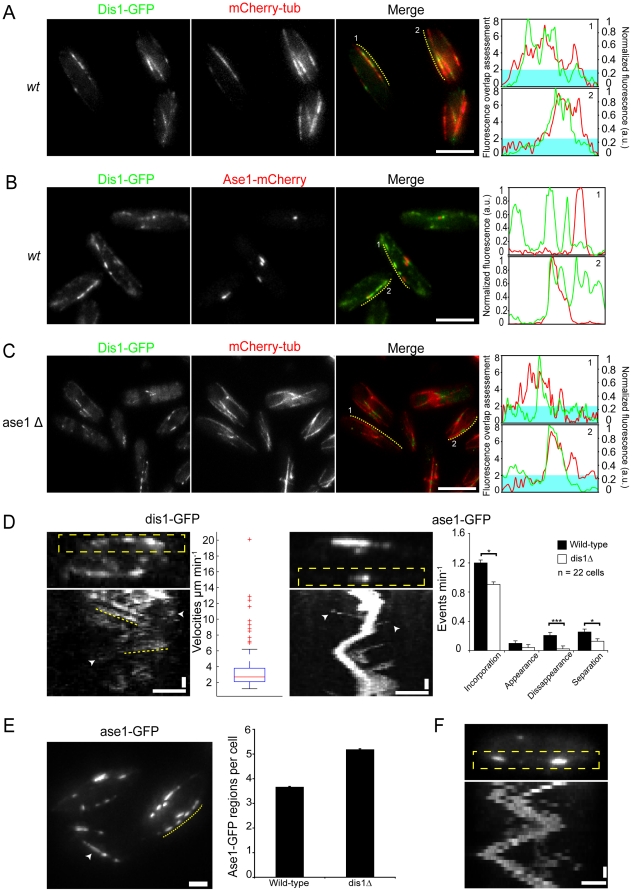
Dis1p-GFP localizes differently to MT overlaps in wild-type and *ase*1Δ cells. A. Wild-type cells labeled with dis1p-GFP and mCherry-Tubulin. Fluorescence intensity profiles of selected IMAs showing that Dis1p-GFP does not localize to the whole overlap regions of mCherry-tubulin (regions of the red line above the turquoise shade). B. Wild-type cells labeled with dis1p-GFP and ase1p-mCherry and intensity profiles of selected IMAs showing that dis1-GFP and ase1-mCherry have both co-localized and segregated signals in different IMAs. C. *ase1Δ* cells labeled with dis1p-GFP and mCherry-Tubulin and intensity profiles of selected IMAs where Dis1p-GFP localizes to the overlap regions of mCherry-tubulin. D. Kymograph showing the dynamics of dis1p-GFP along an IMA (left) where dotted lines indicate different velocities movements. Speckles of dis1p that appear and disappear are visible (arrowheads). The velocities distribution is represented in the box-plot graph. Kymograph of ase1-GFP (center right) along time. Several events of incorporation of newly created overlaps are visible (arrowheads). Graph (right) displaying several events of ase1-GFP overlaps. *p<0.05; ***p<0.001. E. Ase1p-GFP cells over-expressing dis1p. Several regions of ase1p-GFP are visible along the same IMA (dotted line) as well as faint long regions of ase1p-GFP along an IMA (arrowhead). The average number of ase1p-GFP regions per cell is depicted in the bar graph. F. Kymograph showing highly dynamic ase1p-GFP regions in cells over-expressing dis1p. Compare this kymograph with the ase1-GFP wild-type in panel D. Cell images are maximum projections. Bars: 5 µm in A to C; 2 µm in D, E and F. Vertical bars: 30 seconds.

The presence of MT overlap regions in *ase1Δklp2Δ* cells prompted us to search for alternative proteins involved in bundle organization. Such a protein should localize to IMAs similarly to ase1p [22]. Dis1p was identified as one potential candidate as it was reported to bind along the entire length of interphase MTs and to accumulate in medial regions of IMAs [35]. We analyzed this localization in more detail in cells co-expressing a GFP-tagged dis1p variant (dis1p-GFP) and mCherry-tagged alpha-tubulin (mCherry-atb2) [51]. We found on average 2.27 regions of brighter dis1p-GFP fluorescence along the IMAs per cell (n = 66) (Figure 2A). In addition the brighter dis1p-GFP regions did not fully occupy the MT overlap regions (Figure 2A). Analyzing dis1p-GFP in cells expressing mCherry-tagged ase1p (ase1p-mCherry) [26] we observed several intensity peaks of dis1p-GFP along individual IMAs (2.5±0.2, SEM, n = 35) while ase1p-mCherry fluorescence occurred in a single peak (1.42±0.1, SEM, n = 35) (Figure 2B). In order to verify that the labels did not affect protein localization we repeated the same analysis with cells expressing dis1p-tdTomato and ase1p-GFP and obtained similar results (Figure S2A). We confirmed that dis1p-GFP did not fully occupy overlap regions by co-expressing dis1p-tdTomato and cls1p-3GFP. The latter co-localizes with ase1-GFP [26] marking the overlap regions (Figure S2B).

We next analyzed dis1p-GFP localization in *ase1Δ* cells expressing mCherry-atb2. In these cells the number of IMAs with regions of brighter dis1p-GFP fluorescence was reduced (on average 1.8 per cell; n = 61 cells), which is consistent with the expected reduction of MT bundling. The brighter dis1p-GFP regions often coincided with the brighter regions of mCherry-atb2 fluorescence but did not fully occupy the overlap region (Figure 2C). Contrary to the wild-type, dis1-GFP localized mostly in a single peak along the IMA (1.52±0.1, SEM, n = 42) similar to what was observed for ase1-mCherry in wild-type cells. Taken together these results support a role for dis1p in bundle organization.

### The dynamics of dis1p and ase1p differ

For further comparison of dis1p and ase1p we analyzed the dynamics of protein localization in MT overlap regions. We created kymographs of dis1-GFP expressing cells to assess dis1p-GFP dynamics in the IMA. Overall dis1p-GFP was very dynamic, lacking the clearly defined and stable region of MT overlap observed for ase1p-GFP (Figure 2D). Furthermore, speckles of dis1p-GFP were seen to move towards the cell ends, and often appeared or disappeared in the duration of the movie. These data suggest that dis1p binds and unbinds MTs very dynamically (Figure 2D). Nonetheless, some stable regions of increased dis1p-GFP fluorescence were observed and their dynamics quantified (materials and methods). The velocity distribution was comparable to rates of MT growth and shrinkage, indicating that the movement of dis1p-GFP regions could be a direct consequence of MT dynamics (Figure 2D). The movement of dis1p-GFP regions in *ase1Δ* cells was similar to that in wild-type cells but the number of regions with increased fluorescence was significantly reduced (Figure S2C).

We next analyzed the dynamics of ase1-GFP segments on MT bundles in wild-type, *dis1Δ* and over-expressing dis1p cells. In wild-type *and dis1Δ* cells we observed a similar average region displacement velocity of 0.26±0.04 µm minute^−1^ (SEM, n = 50 and 40 respectively) (Figure 2D). In addition we quantified the frequency with which several events occurred in ase1p-GFP regions, namely the merging of distinct regions, the splitting of an ase1-GFP region into smaller regions and the appearance (without incorporation) or disappearance of a region. This analysis revealed that events of incorporation, disappearance and separation were all significantly reduced in *dis1Δ* cells (∼25%, ∼88% and 50%, respectively) (Figure 2D) suggesting that the stability of ase1p-GFP regions is increased in the absence of dis1p. We then investigate whether dis1p over-expression led to changes in the stability of ase1p-GFP regions. These cells contained an average of 5.19±0.02 (SEM) regions of ase1p-GFP per cell (an increase of 41% compared to 3.67±0.016 [SEM] in wild-type cells) with several regions per IMA (Figure 2E). In addition, these regions localized throughout the cell and faint ase1-GFP regions could be seeing along IMAs. Finally, the average displacement velocity was of 1.87±0.01 µm minute^−1^ (SEM, n = 16) (Figure 2F). These results indicate that dis1p affects the localization and dynamics of ase1p.

### Dis1p influences MT bundling

**Figure 3:**
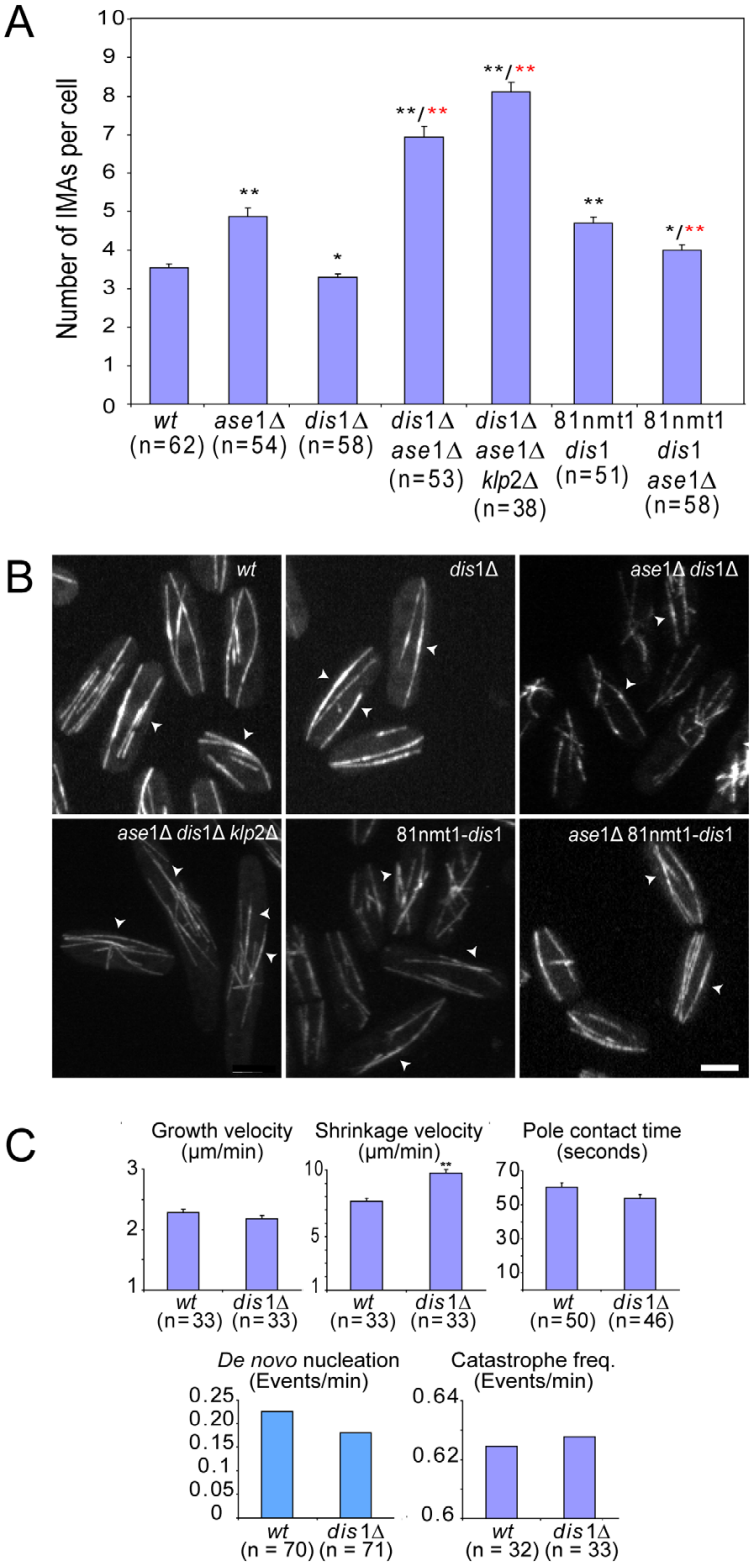
Dis1p over-expression rescues the *ase1Δ* phenotype. A. Number of IMAs per cell for wild-type and the different mutants. * p<0.05; ** p<0.001 (mean ± SEM). Black asterisk are in relation to wild-type and red asterisks to *ase1Δ*. B. GFP-tubulin labeled cells show that the triple mutant *ase1Δdis1Δklp2Δ* has more IMAs than wild-type or any other mutant. Regions of higher fluorescence intensity indicative of MT overlaps (arrowheads) are also visible. Over-expression of dis1p-GFP leads to the rescue of the *ase*1Δ phenotype. C. *dis*1Δ cells do not affect *de novo* nucleation or MT dynamics with the exception of the increase in shrinkage velocity (mean ± SEM). Bar: 3 µm in B.

The observed dis1p localization supported the possibility that this protein contributes to the formation or stabilization of MT bundles. We further investigated this behavior by imaging live cells expressing GFP-tubulin in which *dis1* (*dis1Δ*), *ase1* and/or *klp2* were deleted. In *dis1Δ* cells we detected an 8% decrease in the number of IMAs (Figure 3A) and an 18% decrease in IMA regions with increased fluorescence (Table 1 and Figure 3B) with no significant difference in *de novo* nucleation between wild-type and *dis1*Δ (Figure 3C and Figure S3). In addition the MT shrinkage velocity was increased by 28% (Figure 3C). We analyzed *ase1Δdis1Δ* cells, which showed the largest defects in the positioning of the division plane and were the most sensitive to the MT depolymerizing drug MBC (Figure S4). *Ase1Δdis1Δ* cells showed a 95% increase in the number of IMAs, which is significantly greater than that observed in *ase1Δ* or *ase1Δklp2Δ* cells (Figures 3A and 3B). The MT cytoskeleton appeared disorganized in these cells. IMAs aligned poorly with the long cell axis and were often asymmetrically distributed within the cell (Figure 3B). A few short regions with increased IMA fluorescence were also present in *ase1Δdis1Δ* cells suggesting that some MT bundling is still present (Table 1 and Figure 3B). One possibility is that these regions are stabilized by the third proposed MT bundler klp2p. To test this hypothesis we created GFP-tubulin expressing cells in which *ase1*, *klp2* and *dis1* were deleted (*ase1Δklp2Δdis1Δ*). IMAs in these cells were organized similarly to those in *ase1Δdis1Δ* cells (Figure 3B). The number of IMAs increased by 138%, which is the highest value amongst the mutants we analyzed (Figure 3A and 3B). However, regions containing increased IMA fluorescence were still detectable (a reduction of 57% to the wild-type) (Figure 3B and Table 1).

We next tested the effect of dis1p over-expression on MT bundling in a variety of backgrounds. In the wild-type background this resulted in a 13% increase of the number of regions with enhanced IMA fluorescence (Table 1), suggesting an increase in MT bundling (Figure 3A and 3B and Table 1). The over-expression of dis1p in *ase1Δ* cells did not affect the number of IMA regions with increased fluorescence present in those cells (Figure 3A and 3B). However, the number of IMAs was reduced by 18% compared with *ase1Δ* cells. These results indicate a direct effect of dis1p in MT bundling but also show that the effects of dis1p over-expression are strongly influenced by the presence of ase1p suggesting some degree of redundancy between the activities of the two proteins.

### ET supports role of dis1p in MT bundling

**Figure 4:**
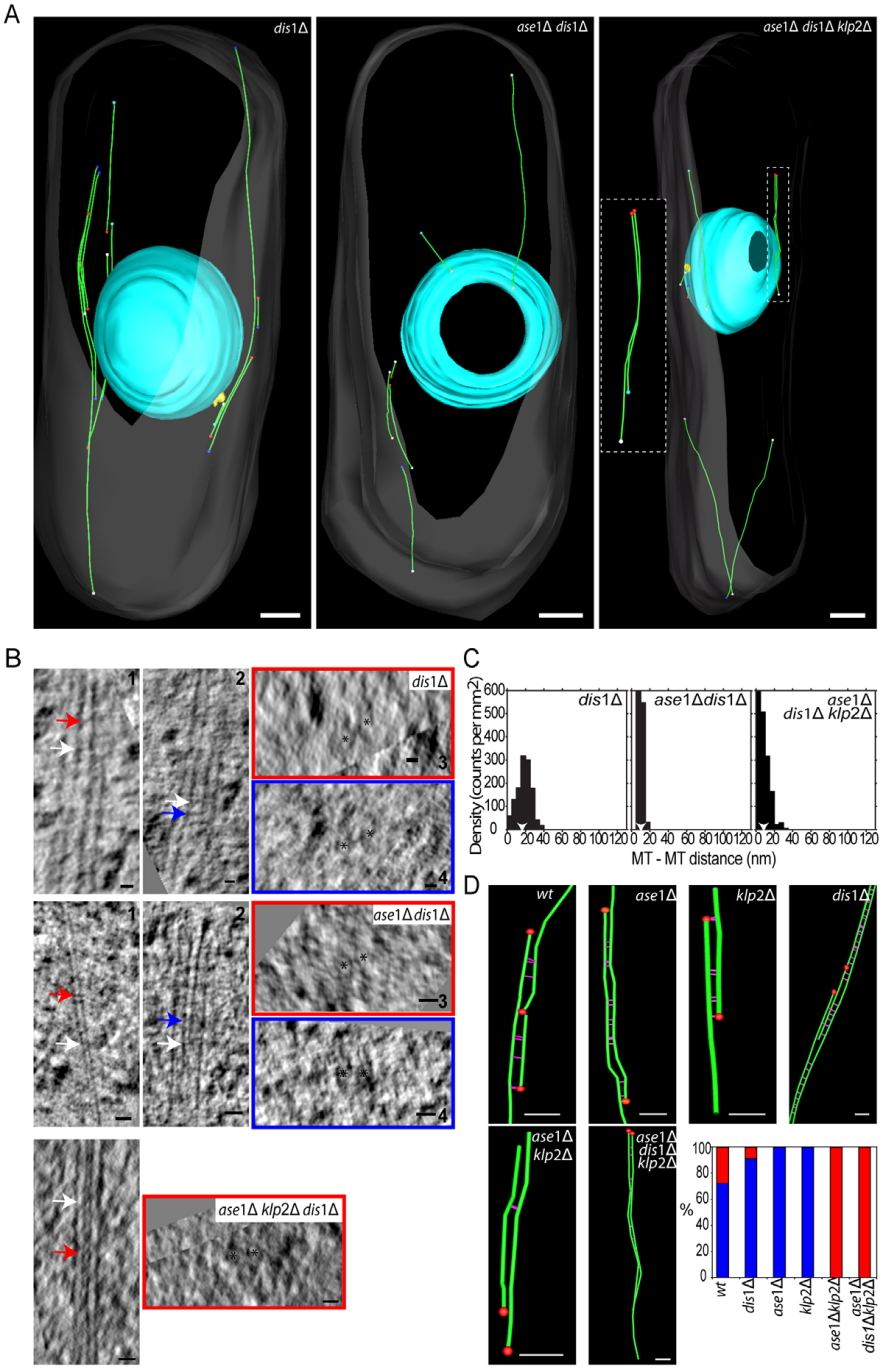
*ase1Δ dis1Δklp2Δ* cells have MT overlap regions. A. Models obtained from selected cells that are representative of each mutant cytoskeleton organization. In the triple mutant the inset shows the MT bundle of the dotted square isolated and rotated for better view. MT bundles are associated or non-associated with the SPB (yellow volume). See also Figure S1B and Movies S4–S6. B. Selected longitudinal tomographic sections of two adjacent MTs (asterisks) (1 and 2) showing electron-dense bridges (arrows). Cross-section of 1 and 2, respectively, in the plane of red and blue arrows. C. Nda analysis show a small decrease in the inter-MT distance for *dis*1Δ cells, while *ase*1Δ *dis*1Δ cells have a significant reduction of the inter-MT distance (arrowheads indicate the mean of the peak). D. Gallery of bundles showing inter-MT associations (100 nm stretches of MT that are at (or closer than) the higher peak values of the Nda analysis to an adjacent MT). MT minus end structure is represented by a red sphere. Percentage of anti-parallel (blue) and parallel (red) MT associations. Bars: 500 nm in A; 25 nm in B; 100 nm in D.

To gain further insight into the proposed role of dis1p in MT bundling, we visualized IMAs in *dis1Δ*, *ase1Δdis1Δ* and *ase1Δklp2Δdis1Δ* cells using ET. In *dis1Δ* cells only bundled MTs were present, confirming that dis1p is not essential for interphase MT bundling (Figure 4A and Table 2). Bundled MTs were connected with electron-dense bridges, but the average inter-MT distance was reduced by 40% compared to wild-type cells, with a peak at 15–25 nm (mean at 16.3 nm) (Figure 4B and C).

In contrast to the single mutants, *ase1Δdis1Δ* cells had a strongly reduced number of MT bundles (Figure 4A and Table 2). Most MTs were unbundled and only two bundles each containing two MTs were found in the reconstructed cell volumes (see Figure S1B). These MTs were connected by electron-dense bridges (Figures 4B), but the inter-MT distance was reduced, similar to *ase1Δ* cells, with a peak at 5–15 nm (mean at 10.2 nm) (Figure 4C).

MT bundling in *ase1Δklp2Δdis1Δ* cells was affected similarly to *ase1Δdis1Δ* cells (Figure 4A and Table 2). Most MTs were isolated, but we found one MT bundle in each volume, one of which consisted of four MTs (see Figure S1B). The inter-MT distance was further reduced compared to *ase1Δdis1Δ* cells, peaking at 0–15 nm (mean = 8.2 nm, a reduction of 69% compared to wild-type) (Figure 4C). Due to the small inter-MT distance it was difficult to identify electron-dense bridges. However in overlaps separated by greater distances such bridges were clearly present (Figure 4B). Our ET results provide high-resolution images, largely confirming the fluorescence imaging data and thus further supporting that dis1p contributes to interphase MT bundling.

### Dis1p interacts with parallel and anti-parallel bundled MTs

To assess whether dis1p has a preference for bundled MTs with parallel or anti-parallel orientation, we determined MT polarity in the tomograms of mutant cells. We analysed MT end structures and distributed them into four categories: capped, blunt, open and ambiguous (Figure S1A). Capped ends are considered to represent MT minus ends bound by γ-tubulin and its associated complex [52]. The presence of a capped end allowed us to assign a polarity to the respective MT. Since the individual MTs in a bundle can interact laterally with several other MTs, we also analyzed the lateral association of MTs with their immediate neighbors. MTs were considered to bundle if they were in close proximity to each other along a 100 nm segment (only bundled MT regions were considered). The proximity threshold was determined by the inter-MT distance peak calculated from Nda analysis (e.g., this sets the upper limit to 35 nm for wild-type cells) (see materials and methods). Each MT could be associated with more than one MT and with differently orientated MTs.

We first analyzed the MTs in previously acquired wild-type cell volumes [9]. In these cells, 72% (24 measured MT segments in 4 bundles) of the bundled MTs with known polarity had an anti-parallel orientation with the remainder orientated in parallel (Figure 4D). In *dis1Δ* cells the percentage of MT segments bundled in an anti-parallel fashion increased to 92% (43 MT segments in 3 bundles measured) suggesting that dis1p promotes parallel MT bundling. Consistently, the few bundled MT segments we identified in *ase1Δklp2Δ* cells (3 MT segments in one bundle) were oriented in parallel. This was also the case in *ase1Δklp2Δdis1Δ* cells (16 MT segments in one bundle). Surprisingly, mutants of ase1p or klp2p, which are both thought to promote anti-parallel MT bundling, did not reduce the number of MTs with anti-parallel orientation but rather increased it to 100% (21 and 8 MT segments in 3 and 2 bundles measured respectively). Although the number of MT orientations that can be determined using ET is too small to provide reliable statistical estimates, these results suggest that dis1p may promote the bundling of MTs with parallel orientation, and that the mechanisms leading to MT bundling may be more complex than is currently appreciated.

## Discussion

In this work we investigated the role of several proteins in the formation of IMAs in *S. pombe*. By combining fluorescence with ET imaging we showed that the formation of IMAs is a complex process involving several proteins (see below). Several lines of evidence indicate that dis1p, one of the two fission yeast XMAP215 homologues, also contributes to this process. Ase1p and dis1p functions appear to be partially redundant since cells lacking both proteins are strongly impaired in their IMA formation, whilst this is only mildly impaired in either of the single deletion mutants. The phenotype arising from deletion of dis1p might be a consequence of dis1p cross-linking or stabilizing MTs either directly or through the recruitment of other proteins. These functions are also consistent with the localization of dis1p to spindle microtubules during anaphase B. Mutations of dis1p that lead to abolition of dis1p binding to interpolar microtubules result in spindles that breakdown earlier than in wild-type [36]. Our results also provide further support for the role of the kinesin-14 motor klp2p in proper IMA formation. In the absence of klp2p, MT overlaps that form away from the medial region of the bundle are not relocated to the center [23], [53], which could prevent stabilization of the daughter microtubule. This model would then explain the small reduction in the number of regions of increased fluorescence and the reduction in the number of MTs per bundle. The residual IMAs present in the *ase1*Δ*dis1*Δ*klp2*Δ triple mutant cells suggest that yet other factors contribute to IMA formation. Other proteins such as the TACC protein family member mia1p/alp7p, which was recently proposed to crosslink MTs at the edge of overlap regions may contribute [21]. It has also been shown that *in vitro* MTs can spontaneously organize into parallel arrays with mixed orientations [54]. Such an intrinsic bundling property would be aided in living fission yeast cells by the fact that new MTs preferentially nucleate on existing ones thus naturally bringing MTs into close proximity with each other [23].

Another possibility is that the second fission yeast XMAP215 homolog, alp14p, could act redundantly with dis1p function [32], [55]. Although alp14p localizes mainly to growing plus ends of interphase MTs (D. Brunner, unpublished), it is possible that in *dis1*Δ cells the protein can access binding sites that are normally occupied by dis1p thereby promoting IMA formation. Similarly, any of the dimeric fission yeast kinesin motor proteins or any other protein with more than one MT binding domain can in principle promote MT crosslinking and stabilization leading to IMA formation in the absence of dis1p/ase1p/klp2p.

An important point to consider in the context of anti-parallel MT bundling is that as soon as more than two anti-parallel MTs are present in an overlap region, two potential stretches of parallel overlap will also be created. These regions could further stabilize the bundle but could also provide competition with ase1p thus weakening anti-parallel MT interactions. The fact that *ase1*Δ*klp2*Δ cells contained MT pairs that are exclusively parallel suggest that dis1p may preferably localize to adjacent MTs with parallel orientation, however, the relatively small number of bundles that can be reconstructed using ET means that this result must be treated with caution. A preference for cross-linking microtubules with a parallel orientation would also explain the localization of dis1p-GFP during anaphase B to the poles of the spindle [36], and could also account for the incomplete co-localization of dis1p and ase1p. Another possibility is that dis1p could be competing with ase1p for binding sites on the MT lattice but with lower affinity. This would explain both the increased/decrease stability of ase1-GFP regions in *dis1Δ* and dis1p over-expressing cells, respectively. In *dis1*Δ cells ase1p has more free binding sites leading to more stable overlap regions with a greater proportion of anti-parallel MT overlaps. In cells over-expressing dis1p, ase1p binding is reduced by the dynamics of dis1p leading to ase1p-GFP being more dynamic and dispersed in the IMAs. Reduced binding of ase1p to MT bundles decreases binding of cls1p, a MT rescue factor involved in maintaining MT overlap regions [26]. Reduced rescue events would increase MT loss. Consequently, IMAs would be less stable and contain fewer MTs on average, while new IMAs would form more frequently. As a result the overall number of IMAs would increase. This model also explains how, in the absence of ase1p, dis1p over-expression causes a reduction in the number of IMAs in these cells by stabilizing or bundling adjacent MTs.

Another so far neglected characteristic of interphase MT bundling is the impact of the bundling proteins on the distance between MT lattices. Differential regulation of inter-MT spacing could in principle offer an intriguing mechanism to control the binding of MT-regulating MAPs and motor proteins. Our results reveal variation of the inter-MT spacing between the various mutant combinations analyzed. While the effect on inter-MT spacing appears to be moderate in *dis1*Δ or *klp2*Δ cells, *ase1* deletion leads to a significant reduction in the spacing between MT walls. This suggests that ase1p is the main determinant of the distance between bundled interphase MTs in wild-type cells. The presence of MT pairs with virtually no inter-MT distance in *ase1*Δ*dis1*Δ*klp2Δ* triple-deleted cells suggests that MT bundling can occur in the absence of an intercalating MT cross-linker. Further experiments are necessary to determine in detail the effect on inter-MT distance of each individual protein and how this might affect the function other MAPs.

Our ET results show the importance of high resolution imaging for an adequate interpretation of fluorescence imaging data, especially when mutant strains and the role of the respective proteins are analyzed. Our data also make clear that MT bundling is a complex process that is achieved by multiple potentially cooperative and antagonistic bundling and cross-linking activities.

## Materials and Methods

### Strains and Preparation

Standard *Schizosaccharomyces pombe* genetic and molecular biology techniques, as well as media, were used as described in the “Nurse Lab Manual” (http://biosci.osu.edu/~nile/nurse_lab_manual.pdf).

Strains and plasmids used in this study are listed in Text S1. Cells were grown and imaged in minimal medium EMM2 supplemented with amino acids (final concentration of 250 mg/L) when necessary and with thiamine to a final concentration of 15 µM. For image acquisition the cells were collected and placed in a 35 mm glass-bottom culture dish (MalTek Corp., Ashland, MA, USA).

Over-expression of dis1p was achieved by performing a pre-culture followed by culture, always in the absence of thiamine. This lead to an over-expression level of ∼4× the endogenous levels of dis1p as determined by fluorescence intensities comparison (Figure S1C). 81nmt1-GFP-dis1p cells and dis1p-GFP cells were simultaneously imaged by labeling the latter with lectin-TRICT. Levels of fluorescence were averaged for more than 50 cells of each strain.

### Fluorescence Live Cell Imaging and analysis

Confocal images were generated using a Carl Zeiss Axiovert 200 M microscope equipped with a PerkinElmer RS Dual spinning disc system. The Argon Krypton line laser was used at wavelength of 488 nm for GFP signal detection. Images were collected using a 100× oil immersion objective (Plan Fluor, NA 1.4) in a Hamamatsu Orca ER camera (Hamamatsu, Japan) with a pixel size of 6.45 µm and analyzed with the Ultraview acquisition software (Perkin Elmer, Foster City, CA US). Z-stacks were taken with 13–16 planes per stack with a distance of 0.5 µm between planes. For dual color images, a Carl Zeiss Axiovert 200 M widefield system was used in combination with a mercury lamp. Images were collected with a Coolsnap HQ camera (Roper Scientific) with a pixel size of 6.28 µm and with a 100× oil immersion objective (Plan Fluor, Na 1.4). Z-stacks were collected with 9–11 planes per stack, with a distance of 0.5 µm between planes. Signal for GFP and mCherry were collected sequentially for each Z plane. All experiments were carried out at room temperature (24–26°C). The assessment of overlap regions was performed by dividing each IMA normalized pixel value by the average of the lower intensity regions (corresponding to one MT). A stretch of more than two pixels with values above two were considered to correspond to two or more MTs. Analysis was performed in maximum projection images. IMAs that overlap other IMAs where not included in the analysis.

### Specimen preparation for Electron Microscopy

Log-phase cultures grown at 25°C of wild-type and mutant strains were collected by filtration and high-pressure frozen using a LEICA EMPACT2 device. Fixation was performed during freeze substitution in dry-acetone containing 0.1% dehydrated glutaraldehyde, 0.25% uranyl acetate and 0.01% osmium tetroxide (OsO_4_). Substitution was initiated at −90°C for 56 hr before the temperature was raised by 5°C *per* hour to −45°C. Plastic infiltration and polymerization was then performed as previously reported [9].

Serial semi-thick sections (250–300 nm) were cut (Leica Ultracut UCT microtome), collected on Formvar coated slot grids and post-stained with 2% uranyl acetate in 70% methanol and Reynold's lead citrate. Cationic 15 nm gold particles (Brittish Bio Cell) were attached to both sides of the grid for use as fiducial markers.

### Electron Tomography

ET was performed essentially as described in Höög, *et al* (2007). Briefly, specimens were placed in a tomography specimen holder (Model 2020, Fischione Instruments, Corporate Circle, PA; or Model 650; Gatan, Pleasanton, CA). Random cells, between 6 and 9 µm in length, orientated perpendicularly to the tilt-axis were selected by visual inspection of the grids. Montage tomographic datasets were collected using a FEI Tecnai TF20 or a FEI Tecnai TF30 at a magnification of ∼14,500× or ∼15,500×, respectively, using the tilt-series acquisition software SerialEM [56]. Images were acquired every 1° over a±65° range using a Gatan 4K×4K CCD camera. Images were aligned using the fiducial marker positions. Tomograms were computed using the R-weighted back-projection [57] and joined in the eTomo graphical interface [58].

### Modeling and Analysis of Tomographic Data

The IMOD software package [59] was used to display, model and analyze tomograms and models. Relevant structures were modeled as reported in Höög *et al* (2007). Microtubules were tracked and their end morphology analyzed using the “slicer” tool. MT ends were marked in different colors to allow distinction [9], [52]. Volumes were extracted from models using the Imodinfo program. The Nda program was use to calculate the distance between MTs and the mean of the major distribution peaks, after converting the model to resemble a model from serial cross-sections, using the Resamplemod program [60]. The program Mtk [61] was used to measure MT associations. In this analysis each MT was split in 100 nm fragments and for each fragment the closest MT fragment was calculated and marked with lines connecting the closest neighbor.

## Supporting Information

Text S1 Supplementary information containing the list of strains used in this work.
(0.05 MB DOC)

Figure S1 MT end morphologies and *ase1 dis1* deleted and *ase1 dis1 klp2* deleted bundles related to Figures 1 and 4. A. Micrograph, graph and color scheme of the different types of MT end morphology used to determine MT orientation. Color circles refer to color spheres used in the models to mark MT end morphology. From left to right: blunt end; open end; and capped end. Undetermined MT ends were marked with white spheres. MT orientation was only determined if a capped end was present. Bar: 25 nm. B. One of the two bundles found in the *ase1 dis1* deleted volumes and the bundle with four MTs in the *ase1 dis1 klp2* deleted volume. Bars: 250 nm. C. Gallery of cells used for dis1p over-expression assessment. Graph showing the average difference of fluorescence expression. Black bars represent average background values for each strain.
(1.96 MB TIF)

Figure S2 Co-localization of dis1p with MT overlaps. A. Co-localization of dis1p-tdTomato and ase1p-GFP in wild-type cells. Similar results were obtained with this set of labels as the ones obtained with dis1p-GFP and ase1-mCherry. B. Co-localization of dis1p-tdTomato and cls1–3GFP confirms that dis1p partially localizes with MT overlaps. C. Kymographs showing the dynamics of dis1p-GFP and ase1-mCherry along the IMAs of wild-type cells. Dis1p-GFP does not fully localize to the MT overlap regions. In wild-type cells several stretches of higher intensity fluorescence are visible, while in *ase1* deleted cells only two stretches are visible corresponding to two overlap regions. Horizontal bars: 5 µm. Vertical bar: 15s.
(6.06 MB TIF)

Figure S3 MT dynamic analysis of wild-type and *dis1* deleted cells. A. Kymographs of selected IMAs (upper panel dotted area) where are visible events of MT growth (green dotted lines), shrinkage (red dotted lines) and *de novo* nucleation along a pre-existent IMA (arrows). B. Time-lapse imaging of GFP-tubulin expressing cells where *de novo* nucleation occurs in the cytoplasm where no other MTs were previously visible (arrowheads). Horizontal bars: 2 µm. Vertical bars: 30s. Frame delay is 5s in B.
(2.00 MB TIF)

Figure S4 A. Bar graph showing the ratio between the smaller half of a septated cell and cell length at 30 degrees. *Ase1 dis1* deleted cells have the major defects in septum positioning followed by *ase1* deleted cells. Gallery of images representative of each strain (arrowheads indicate septum) where bent cells are visible in the double mutant strain. B. Colony formation assay of different strains with solvent (DMSO) and two increasing concentrations of the MT depolymerizing drug MBC. Again the double mutant strain is the most sensitive to the drug.
(1.60 MB TIF)

Movie S1 Model of a reconstructed *ase1* deleted cell volume.
(6.91 MB AVI)

Movie S2 Model of a reconstructed *klp2* deleted cell volume.
(7.84 MB AVI)

Movie S3 Model of a reconstructed *ase1* and *klp2* deleted cell volume.
(7.21 MB AVI)

Movie S4 Model of a reconstructed *dis1* deleted cell volume.
(6.50 MB AVI)

Movie S5 Model of a reconstructed *ase1* and *dis1* deleted cell volume.
(5.57 MB AVI)

Movie S6 Model of a reconstructed *ase1, dis1* and *klp2* deleted cell volume.
(5.06 MB AVI)
