# Supplementary material for: The Fission Yeast XMAP215 Homolog Dis1p Is Involved in Microtubule Bundle Organization
Source: PLoS One. 2010 Dec 2;5(12):e14201. doi: 10.1371/journal.pone.0014201 (PMC2996303; doi:10.1371/journal.pone.0014201)
Supplement: Text S1 — Supplementary information containing the list of strains used in this work. (0.05 MB DOC) [file pone.0014201.s001.doc]

**Supplementary information**

**Table S1:** List of strains used in this study

| Strain | Genotype | Source |
| --- | --- | --- |
| DB 558 | h- |  |
| DB 1487 | h+ *ase1Δ::KanMX6* *leu1.32, ura4.D18* | S1 |
| DB 707 | h- *klp2-D25::ura4+* *ade6-M210 his3-D1 leu1-32 ura4-D18* | S2 |
| DB 1469 | h-, *dis1-GFP::kanr* | This study |
| DB 1539 | h-, *ase1Δ::kanMX6* *klp2Δ:ura4+ leu1.32* | S3 |
| DB 1197 | h-, *lys1+::nmt1-GFP- α2tub ura4-D18* | ?? |
| DB 1845 | h+, *ase1Δ ::KanMX6 lys1+::nmt1-GFP- α2tub* | This study |
| DB 2082 | h?, *klp2-D25::ura4+ lys1+::nmt1-GFP- α2tub* | This study |
| DB 2094 | h+, *ase1Δ ::KanMX6 klp2-D25::ura4+ lys1+::nmt1-GFP- α2tub* | This study |
| DB 1636 | h-, 81nmt1-GFP-dis1::kanr | This study |
| DB 1724 | h+, *dis1∆::hph* | This study |
| DB 2076 | h-, *leu1-32::pSV40-GFP-atb2[leu1+] 81nmt1-dis1::kanr leu1-32* | This study |
| DB 2265 | h-, *dis1∆::hph* *ase1::KanMX6 ura4-D18 leu1.32* | This study |
| DB 2258 | h+, *dis1∆::hph ase1∆::KanMX6 lys1+::nmt1-GFP-α2tub ura4-D18? leu1-32* | This study |
| DB 2438 | h?, *aur::cherry-atb2 dis1-GFP::KanMX leu1-32? ura4-D18* | This study |
| DB 2439 | h+, *auR::cherry-atb2 ase1Δ::kanMX6 dis1-GFP::KanMX leu1-32? ura4-D18?* | This study |
| DB 2353 | h-, *Dis1-GFP::KanR ase1-mCherry:Nat4=MX4 leu1 ura4-D18 ade6-M216* | Ase1-mCherry from S4 |
| DB 2343 | h?, *ase1::KanMX6 leu1-32::pSV40-GFP-atb2[leu1+] 81nmt1-dis1::kanr leu1-32 ura4-D18?* | SV40-GFP-atb2 from S4 |
| DB 2442 | h-, *dis1∆::hph ase1Δ::KanMX6 klp2Δ::ura4 lys1::nmt1-GFP-alpha2tub ura4-d18? leu1-32?* | This study |
| DB 2473 | h-, *dis1∆::hph ase1Δ::KanMX6 klp2Δ::ura4 ade6-M210 ura4-D18* | This study |
| DB 2867 | h?, *ase1-GFP::kanMX6 dis1-tdTomato::kanR ura4.D18* | This study |
| HR1 | h?, *dis1-tdTomato::kanR cls1-3GFP::kanMX ura4-d18? leu1-32? ade6M-216?* | This study |

**Supplementary references**

S1. Loiodice, I., Staub, J., Setty, T.G., Nguyen, N.P., Paoletti, A., and Tran, P.T. (2005). Ase1p organizes antiparallel microtubule arrays during interphase and mitosis in fission yeast. Mol Biol Cell *16*, 1756-1768.

S2. Troxell, C.L., Sweezy, M.A., West, R.R., Reed, K.D., Carson, B.D., Pidoux, A.L., Cande, W.Z., and McIntosh, J.R. (2001). pkl1(+)and klp2(+): Two kinesins of the Kar3 subfamily in fission yeast perform different functions in both mitosis and meiosis. Mol Biol Cell *12*, 3476-3488.

S3. Janson, M.E., Loughlin, R., Loiodice, I., Fu, C., Brunner, D., Nedelec, F.J., and Tran, P.T. (2007). Crosslinkers and motors organize dynamic microtubules to form stable bipolar arrays in fission yeast. Cell *128*, 357-368.

S4. Bratman, S.V., and Chang, F. (2007). Stabilization of overlapping microtubules by fission yeast CLASP. Dev Cell *13*, 812-827.
