# Supplementary figures and images for: The Fission Yeast XMAP215 Homolog Dis1p Is Involved in Microtubule Bundle Organization
Source: PLoS One. 2010 Dec 2;5(12):e14201. doi: 10.1371/journal.pone.0014201 (PMC2996303; doi:10.1371/journal.pone.0014201)

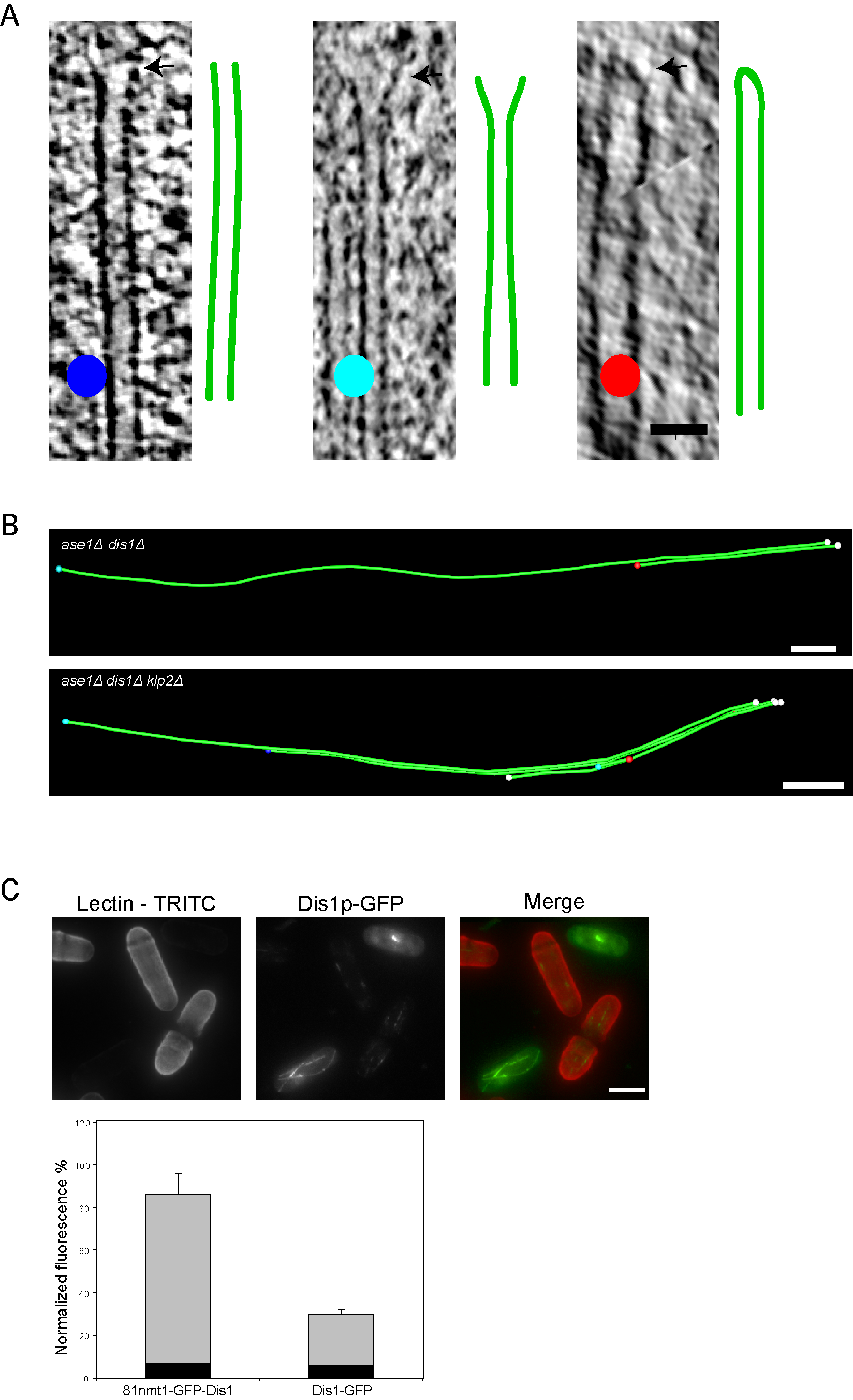

Supplement: Figure S1 — MT end morphologies and ase1 dis1 deleted and ase1 dis1 klp2 deleted bundles related to Figures 1 and 4. A. Micrograph, graph and color scheme of the different types of MT end morphology used to determine MT orientation. Color circles refer to color spheres used in the models to mark MT end morphology. From left to right: blunt end; open end; and capped end. Undetermined MT ends were marked with white spheres. MT orientation was only determined if a capped end was present. Bar: 25 nm. B. One of the two bundles found in the ase1 dis1 deleted volumes and the bundle with four MTs in the ase1 dis1 klp2 deleted volume. Bars: 250 nm. C. Gallery of cells used for dis1p over-expression assessment. Graph showing the average difference of fluorescence expression. Black bars represent average background values for each strain. (1.96 MB TIF) [file pone.0014201.s002.tif]

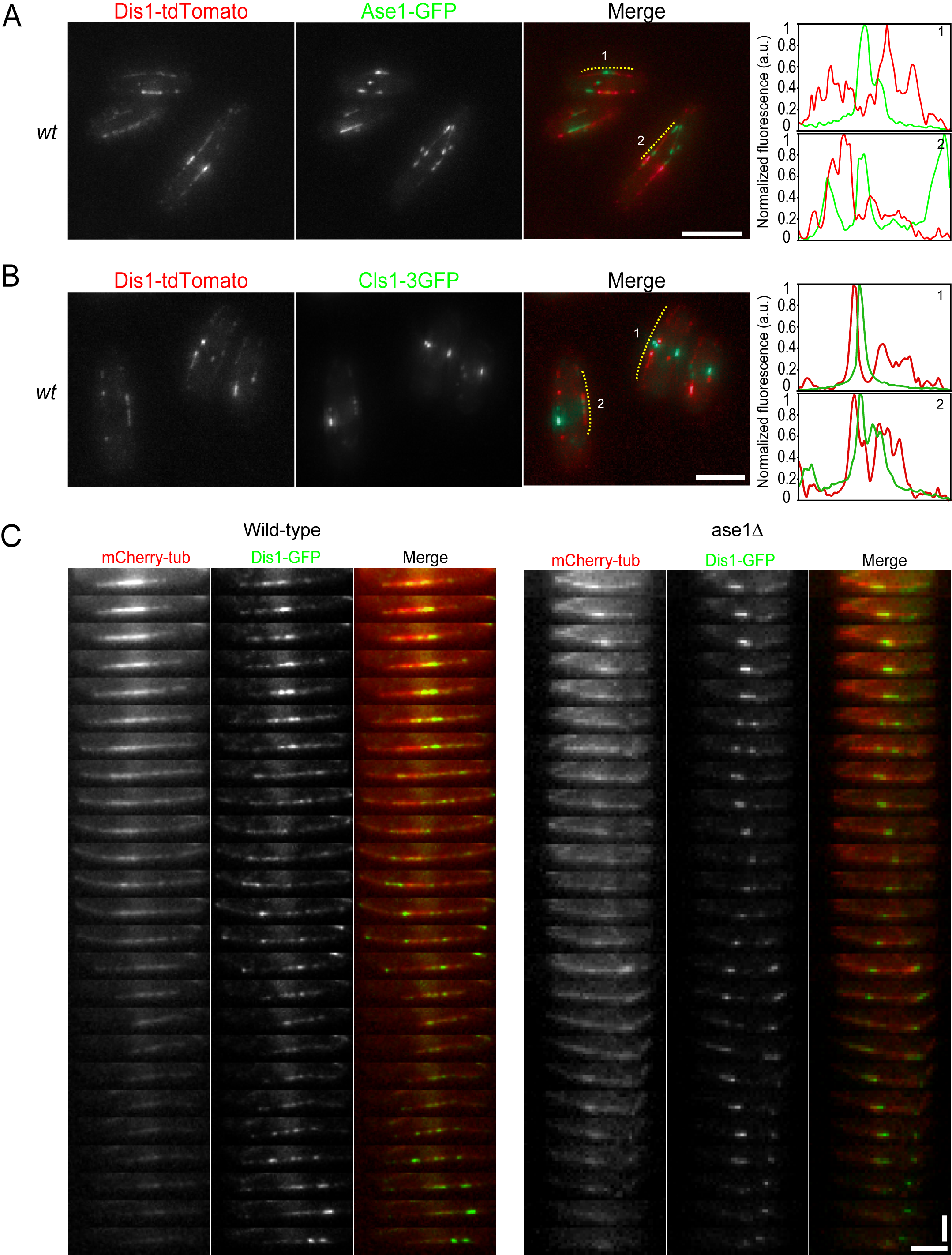

Supplement: Figure S2 — Co-localization of dis1p with MT overlaps. A. Co-localization of dis1p-tdTomato and ase1p-GFP in wild-type cells. Similar results were obtained with this set of labels as the ones obtained with dis1p-GFP and ase1-mCherry. B. Co-localization of dis1p-tdTomato and cls1–3GFP confirms that dis1p partially localizes with MT overlaps. C. Kymographs showing the dynamics of dis1p-GFP and ase1-mCherry along the IMAs of wild-type cells. Dis1p-GFP does not fully localize to the MT overlap regions. In wild-type cells several stretches of higher intensity fluorescence are visible, while in ase1 deleted cells only two stretches are visible corresponding to two overlap regions. Horizontal bars: 5 µm. Vertical bar: 15s. (6.06 MB TIF) [file pone.0014201.s003.tif]

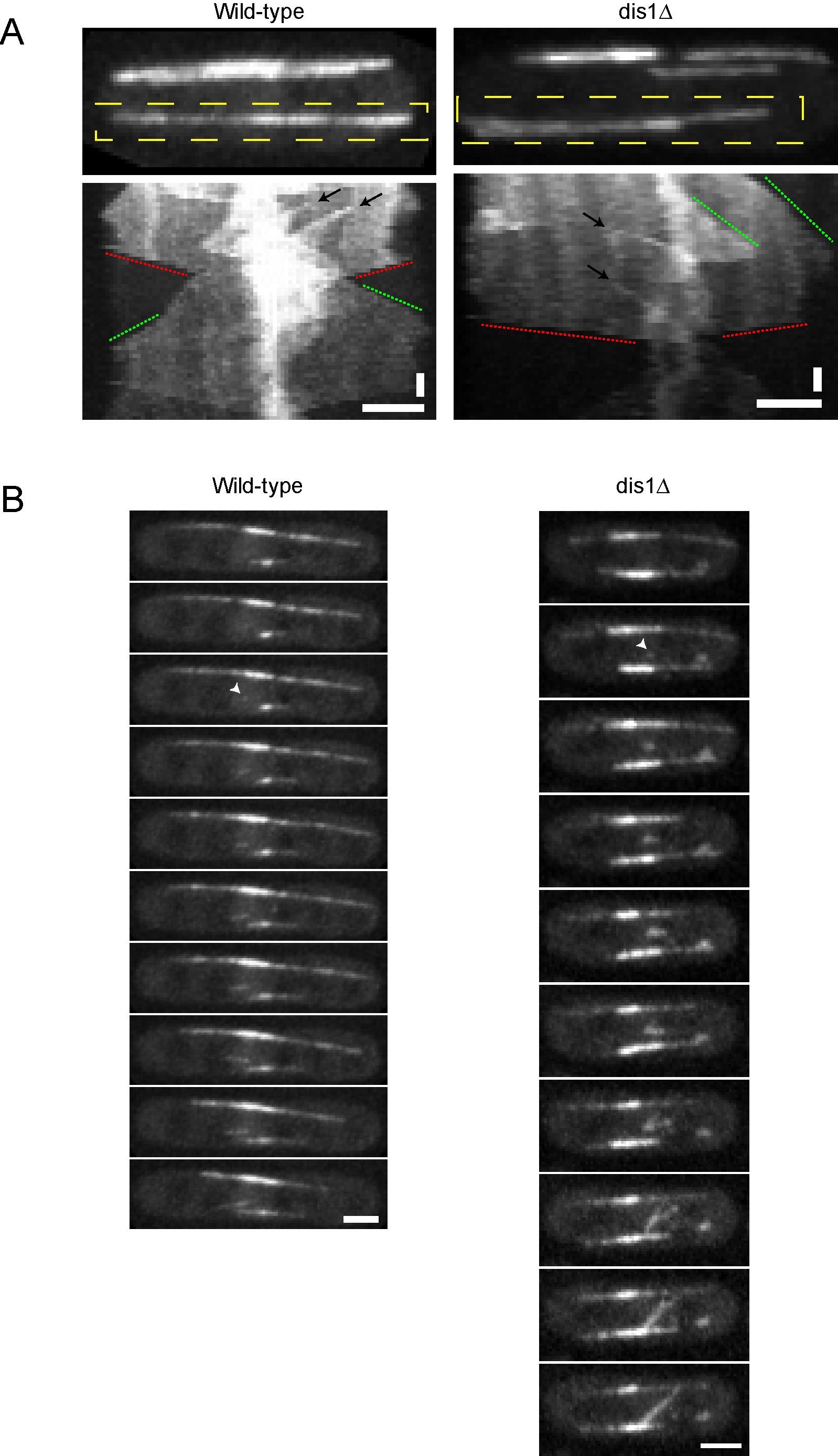

Supplement: Figure S3 — MT dynamic analysis of wild-type and dis1 deleted cells. A. Kymographs of selected IMAs (upper panel dotted area) where are visible events of MT growth (green dotted lines), shrinkage (red dotted lines) and de novo nucleation along a pre-existent IMA (arrows). B. Time-lapse imaging of GFP-tubulin expressing cells where de novo nucleation occurs in the cytoplasm where no other MTs were previously visible (arrowheads). Horizontal bars: 2 µm. Vertical bars: 30s. Frame delay is 5s in B. (2.00 MB TIF) [file pone.0014201.s004.tif]

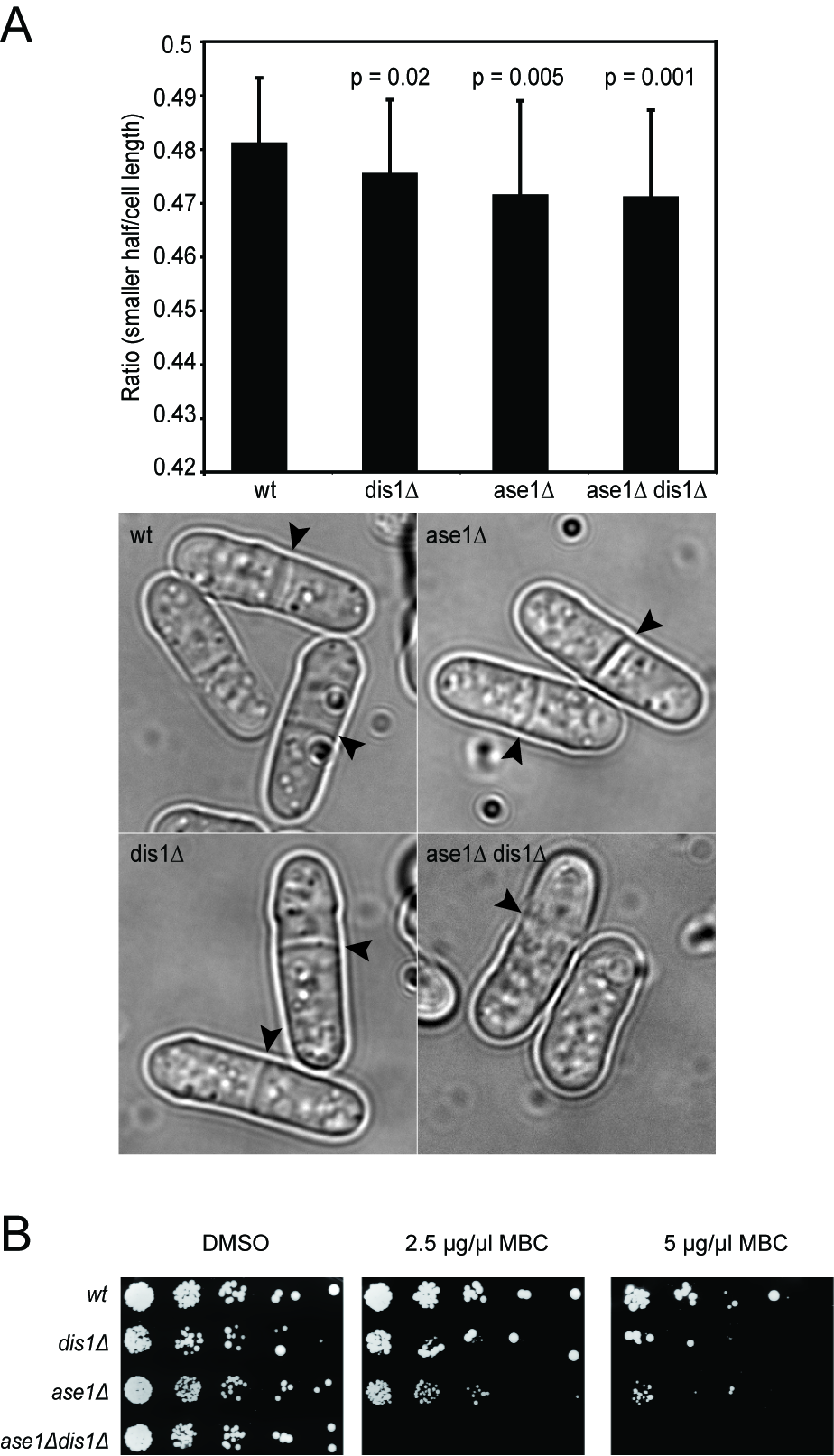

Supplement: Figure S4 — A. Bar graph showing the ratio between the smaller half of a septated cell and cell length at 30 degrees. Ase1 dis1 deleted cells have the major defects in septum positioning followed by ase1 deleted cells. Gallery of images representative of each strain (arrowheads indicate septum) where bent cells are visible in the double mutant strain. B. Colony formation assay of different strains with solvent (DMSO) and two increasing concentrations of the MT depolymerizing drug MBC. Again the double mutant strain is the most sensitive to the drug. (1.60 MB TIF) [file pone.0014201.s005.tif]
